# Evidence for the Direct Effect of the NPFF Peptide on the Expression of Feeding-Related Factors in Spotted Sea Bass (*Lateolabrax maculatus*)

**DOI:** 10.3389/fendo.2019.00545

**Published:** 2019-08-06

**Authors:** Qing Li, Haishen Wen, Yun Li, Zhanxiong Zhang, Yangyang Zhou, Xin Qi

**Affiliations:** Key Laboratory of Mariculture, Ocean University of China, Ministry of Education, Qingdao, China

**Keywords:** spotted sea bass, NPFF/NPFFR system, feeding, *in situ* hybridization, static incubation

## Abstract

Neuropeptide FF (NPFF) is a family member of RF-amide peptides, which are suggested to be involved in the control of vertebrate feeding behavior. However, little is known about the effect of the NPFF peptide on feeding-related processes in basal vertebrates. In this study, four full-length cDNAs, *npff*, *npffr1, npffr2-1*, and *npffr2-2*, were cloned from spotted sea bass and characterized. The conserved NPFF peptide is biologically active because it functionally interacts with different receptors expressed in cultured eukaryotic cells to enhance CRE promoter activity. Tissue distribution analysis showed that the highest *npff* mRNA expression occurred in the telencephalon, hypothalamus, medulla, gonad and muscle, but the *npffr*s mRNAs were mainly distributed within the central nervous system (CNS). *In situ* hybridization (ISH) detected *npff*-expressing cells in several specific regions ranging across the telencephalon and midbrain to the hypothalamus. Incubation of the spotted sea bass conserved NPFF peptide significantly increased the expression of *orexin* (*orx*) and *neuropeptide Y* (*npy*) mRNA and decreased the expression of *leptin* (*lep*), *somatostatin* (*ss*), and *cholecystokinin* (*cck*) mRNA in brain cells. Similarly, the conserved NPFF peptide also heightened the expression of *gastrin* (*gas*), *ghrelin* (*ghrl*), and *motilin* (*mtl*) mRNA and significantly reduced the expression of *cck* mRNA in the intestine and stomach. Taken together, these data suggest that the NPFF peptide may play a stimulating role in regulating feeding-related processes in spotted sea bass.

## Introduction

Neuropeptide FF (FLFQPQRFamide, NPFF) belongs to the RF-amide peptide family (peptides with a C-terminal Arg-Phe-NH_2_) and was first isolated from the bovine brain (1). NPFF is generated from the NPFF precursor peptide, which also produces neuropeptide AF (NPAF) (2). NPFF immunoreactivity was observed in the medial hypothalamus and nucleus of the solitary tract (NTS) in the rat brain (3, 4). A majority of NPFF neurons are located in a zone between the dorsomedial (DMH) and ventromedial (VMH) hypothalamus (5). NPFF was originally found to regulate the analgesic effect of morphine (1); soon afterward, additional physiological functions were discovered in other animals. NPFF and its analogs can effectively lower mouse body temperature (6) after injection. In addition, the roles of NPFF in regulating blood pressure (7), gastrointestinal motility (8), epilepsy (9) and insulin secretion (10) cannot be ignored. Recent studies have shown that NPFF inhibits adipocyte differentiation (11) and increases the M2 activation and self-renewal of adipose tissue macrophages (12) in murine animals and humans.

In humans, NPFFR1 and NPFFR2, which are also referred to as GPR147 and GPR74 in mammals, have been identified as G protein-coupled receptors (GPCRs) and share ~50% identity with each other (13, 14). NPFFR1 binds to RFRP peptides belonging to the RF-amide peptide family, but NPFF preferentially activates NPFFR2 (15). NPFFR1 and NPFFR2 are highly conserved compared with other species, implying that they play critical roles across different species (16). Bonini et al. (13) demonstrated that *NPFFR1* and *NPFFR2* are widely distributed in the central nervous system (CNS) but differ significantly between humans and rats. Many *in vitro* experiments have shown that NPFFR1 plays a role in regulating the reproduction process and inhibiting the forskolin-stimulated accumulation of cAMP by RF-amide peptides (17–22). NPFFR2 is most highly expressed in the hypothalamus, superficial layers of the spinal cord and thalamic nuclei (13, 23, 24) and is involved in activation of the ERK (extracellular signal-regulated protein kinase) pathway (25) and the regulation of pain and sensory input.

Most previous studies have focused on the pharmacological functions of the NPFF/NPFFR system in human and rodents, and there have been few reports on fish or basal vertebrates. A *zfPQRF* gene was cloned in the zebrafish (26) and expressed in the bulbus olfactorius and nucleus olfactoretinalis of the telencephalon but not in the hypothalamus or brain stem. In dwarf gourami (*Colisa lalia*), NPFF can inhibit the pacemaker activity of *TN-GnRH* neurons (27). The *npff* gene was identified in lampreys (28), hagfish (29), and amphioxus (30). Interestingly, three peptides are encoded by lamprey and brown hagfish *npff* precursors; while, the *npff* precursors of other species encode only two peptides (28, 31). The latest report (32) showed that the grass puffer NPFF precursor protein can be separated into a pair of mature peptides and play a possible regulatory role in reproduction. Moreover, in amphioxus, common ancestral forms of the *npff* and *gnih* genes and their receptor genes have been identified (33). All studies on basal vertebrates have focused on the molecular characterization, phylogenetic analysis, and tissue expression patterns of *npff* as described above, but the physiological functions of the NPFF peptide have seldom been studied.

To date, the role of NPFF and its paralog GnIH (RFRP, NPVF, or LPXRFa) in feeding has been reported in several vertebrates. Specifically, food intake was decreased by NPFF (34), while water intake was increased by the same peptide (35). Although injection of lower doses of the NPFF peptide (5–10 nM) can reduce food intake, higher doses of NPFF increase food intake (36). Prior evidence has confirmed that loss of *NPFFR2* in male rats is associated with a significantly decreased food intake (37). On the other hand, gonadotropin-inhibitory hormone (GnIH), a paralog of NPFF, was also proved to regulate food intake via NPFFR1 in chicks and jerboas (38–40). In chicks, intracerebroventricular (ICV) injection of GnIH significantly stimulated food intake mediated by the opioid mu-receptor (38, 39). Likewise, i.c.v injection of RFRP-3 induced a 4-fold increase in food intake in *ad-libitum* female jerboas while simultaneously decreasing *Pomc* levels and increasing *Npy* mRNA levels (40). In summary, based on these lines of evidence, NPFF or GnIH might stimulate feeding behavior via NPFFR2 or NPFFR1 in some vertebrates. Feeding-related processes include appetite control, food intake and gastrointestinal motility. On the other hand, previous studies have shown that the NPFF/NPFFR2 system has a bidirectional regulatory effect on gastrointestinal motility in rats (41, 42). However, the role of NPFF in the fish feeding process remains to be elucidated.

The regulation of ingestion in fish is influenced by many external and internal factors, among which appetite and gastrointestinal motility are key. Studies have shown that most dietary regulatory factors are formed in the hypothalamus. Orexin (Orx) interacts with other dietary regulators, including Neuropeptide Y (Npy) and Leptin (Lep), in regulating the feeding process (43, 44). Furthermore, gastrointestinal hormones such as Gastrin (Gas), Motilin (Mtl), Ghrelin (Ghrl) and cholecystokinin (Cck) have been recognized as important factors influencing the regulation of gastrointestinal motility and appetite (45–47). Spotted sea bass (*Lateolabrax maculatus*) is an important economic aquacultural species in China (48), with its production exceeding 160 thousand tons in 2018. However, most of the aquaculture fish showed serious obesity with substantial visceral fat. To further understand the endocrinology regulation of food intake and energy consumption, we herein used spotted sea bass as a model to address these pivotal issues. After molecularly cloning *npff, npffr1, npffr2-1*, and *npffr2-2*, we investigated their ligand-receptor binding activities and gene expression patterns as well as the cellular localization of *npff* mRNA in the brain. Furthermore, we measured some genes related to appetite control and gastrointestinal motility by incubating brain cells, intestinal tissues and stomach tissues with the spotted sea bass conserved NPFF peptide to evaluate the *in vitro* effects of NPFF on feeding regulation.

## Materials and Methods

### Animals

Spotted sea bass (*n* = 3) with body weights ranging from 1,000 to 1,200 g were purchased from a local fish market in Qingdao, China. All fish were temporarily reared in a plastic bucket at 25 ± 1°C under a 14:10 h light-dark photoperiod and fed commercial feed (Haitong, Fujian, China) twice a day. The spotted sea bass were anesthetized with tricaine methanesulfonate (MS-222, 0.1 g/L) before anatomical assessments. All animal experiments were conducted in accordance with the guidelines and approval of the respective Animal Research and Ethics Committees of Ocean University of China.

### Cloning and Sequence Analysis of *npff* and *npffr*s

Total RNA was extracted from the brain tissues of spotted sea bass using TRIzol Reagent (Invitrogen, USA) according to the manufacturer's instructions. The total RNA concentration and purity were measured using a UV spectrophotometer (ChampGel 5000, China). A 1.5% agarose gel was used to detect RNA integrity. A total of 500 ng of total RNA was used as a template for reverse transcription using a two-step method with a PrimeScript™ RT reagent Kit with gDNA Eraser (Perfect Real Time) (Takara, Japan) according to the manufacturer's protocols. All open reading frame (ORF) sequences were identified in the whole genome sequence database (unpublished data) of the spotted sea bass. ORFs of *npff* and *npffr*s were cloned using the synthesized cDNA. All primers (Table 1) used in this study were designed using Premier 5 software. For PCR, 2×Taq PCR Master Mix (Tiangen, China) was used in the following protocol: denaturation at 94 °C for 3 min; 40 cycles of denaturation for 30 s at 94 °C, 30 s at 55 °C, and 30 s at 72 °C; and a final elongation of 5 min at 72 °C. The PCR product was purified with a TIANgel Midi Purification Kit (Tiangen, China). The final product was subcloned into the PEASY-T1 vector (Tiangen, China), and four different individual positive clones were confirmed by sequencing (The Beijing Genomics Institute, China). All spotted sea bass *npff* and *npffr*s sequences have been submitted to the NCBI database under the following accession numbers: *npff* (MK816461), *npffr1* (MK816462), *npffr2-1* (MK816463), and *npffr2-2* (MK816464).

**Table 1:** Primers used in this study.

**Primers**	**Sequence (5^**′**^-3^**′**^)**
**Primers for ORF cloning**	
*npff*-ORF-F	ATGGACACAGCTGCGGTGGT
*npff*-ORF-R	TTATTTCTTGCCGAATCTCT
*npffr1*-ORF-F	ATGGAGATACTGGACAACGT
*npffr1*-ORF-R	TCAGTTATCCCACGCCTGAT
*npffr2-1*-ORF-F	ATGGACCAGAATCTAATTCC
*npffr2-1*-ORF-R	CTAAATCTGAGACACCTTTTCC
*npffr2-2*-ORF-F	ATGAATGAAGGACTTGGGAA
*npffr2-2*-ORF-R	TCAAATAGACACTGCAGTCAC
**Primers for synthesizing sense**	
**and antisense digoxigenin**	
**(DIG)-labeled riboprobes**	
*npff*-ish-F	CGCATTTAGGTGACACTATAGAA GCGCTGCGGTGGTGACTCTTCTGG
*npff*-ish-R	CCGTAATACGACTCACTATAGGGAGACA ATCCTCCGACATTACCTGCCC
**Primers for real-time PCR**	
*npff*-qRT-F	GCTGCGGTGGTGACTCTTCTG
*npff*-qRT-R	TTGTTCGGACTGCCTTGGATGTG
*npffr1*-qRT-F	GGTCTACACGGCGGTTCT
*npffr1*-qRT-R	CAGCCAGTGGGCGAAA
*npffr2-1*-qRT-F	TATCGCCACCTCAAAGC
*npffr2-1*-qRT-R	TTCAGACCCAACTCCACTC
*npffr2-2*-qRT-F	CTTTCCTGGCTGCCTCTGTG
*npffr2-2*-qRT-R	GCCTTCTCCAGGTCCTCCAT
*orx*-qRT-F	TGCAGAGCCGACTCCACCAG
*orx*-qRT-R	CAGGCAGGAGCGTTGTGATGG
*ss*-qRT-F	GGTGCTTCTTGTGGCTTTG
*ss*-qRT-R	GAGGTCCTTGTCGTTGGTGA
*npy*-qRT-F	GAGGGATACCCGATGAAACCG
*npy*-qRT-R	CCTCTTTCCATACCTCTGTCTCG
*lep*-qRT-F	TGCAACTTTTAAGTGGGGGTA
*lep*-qRT-R	TGTTGTAACCCTCCAGCACGG
*gas*-qRT-F	TGCTAAGAGGGAGAAACTG
*gas*-qRT-R	TATCTCGCGTTCATCGTC
*ghrl*-qRT-F	ACACCTGTTTGCTGGTCTTTC
*ghrl*-qRT-R	ATGTGATGTGGTTGGCCTCTG
*mtl*-qRT-F	TGCTGATGAAGGAGCGAGAA
*mtl*-qRT-R	TCCACCATGTTCCACCTGAG
*cck*-qRT-F	TGCCAACTACAACCAACCT
*cck*-qRT-R	GCGTCGTCCAAAGTCCAT
**Reference gene**	
*18s*-F	GGGTCCGAAGCGTTTACT
*18s*-R	TCACCTCTAGCGGCACAA

The supposed cleavage sites of the NPFF precursor were predicted by Neuropred software, and the mature peptides were forecasted by comparison with other species using Clustal W2 software (http://www.ebi.ac.uk/Tools/msa/clustalo/). Phylogenetic trees were constructed using the neighbor-joining (NJ) method via MEGA 6.0 software. The putative signal peptide of the NPFF precursor was predicted by SignalP 3.0 (http://www.cbs.dtu.dk/services/SignalP/). The transmembrane helical regions of Npffrs were predicted by TMHMM Server 2.0 (http://www.cbs.dtu.dk/services/TMHMM/).

### Cell Culture, Transfection, and Functional Assays

The ORFs of spotted sea bass *npffr*s cDNA were subcloned into pcDNA3.1(+) expression vectors. The spotted sea bass conserved NPFF peptide (LLHQPQRF) was synthesized by GL Biochem (Shanghai, China) at purity exceeding 95%. Prior to transfection, 293-T cells were maintained at 37°C in DMEM (SparkJade, China) supplemented with 10% fetal bovine serum (FBS) (BioInd, Israel). Sixteen hours before transfection, 1 × 10^5^ cells/well were seeded into 24-well tissue culture plates. Then, 1,000 ng of pCRE-Luc reporter plasmids; 1000 ng of pcDNA3.1-npffr1, pcDNA3.1-npffr2-1 or pcDNA3.1-npffr2-2; and 100 ng of pRL-TK (to normalize transfection efficiency) containing Renilla luciferase were transiently cotransfected into the cells in 750 ml of serum-free medium using Xfect^TM^ Polymer (Takara, Japan). Four hours after transfection, the cells were treated with various (from 10^−8^ to 10^−6^ M) concentrations of NPFF for 48 h, and 0 M NPFF was used as a control. Cells were collected, and luciferase activity assays were carried out using a Dual-Luciferase kit (Promega, USA).

### Tissue Distribution

To determine the mRNA levels of *npff* and *npffr*s, telencephalon, hypothalamus, cerebellum, midbrain, medulla, pituitary, head kidney, kidney, spleen, stomach, intestine, gill, heart, gonad, liver, and muscle samples were collected from three adult spotted sea bass. Tissue samples were stored at −80°C for total RNA isolation, reverse transcription PCR (RT-PCR) and qRT-PCR. Primers for *npff* and *npffr*s are listed in Table 1.

### RNA *in situ* Hybridization (ISH)

For detection of *npff* mRNA, a pair of sense and antisense riboprobes for the spotted sea bass *npff* gene were generated using the DIG RNA Labeling Kit (Roche Diagnostics, Mannheim, Germany) as directed by the manufacturer. Brain tissue was collected from adult male spotted sea bass for ISH and then fixed in 4% paraformaldehyde buffer for 24 h. After dewatering by a series of graded ethanol solutions (70–100%) and permeabilization in xylene, the sample was immersed in paraffin. The paraffin was cut into 7-micron slices on glass slides. After hydration and permeabilization, the sample was digested by proteinase K (10 mg/mL) for 20 min and then prehybridized at 55°C for 1 h. The slice was hybridized overnight at 55°C with digoxigenin (DIG)-labeled probes diluted to 0.1% using hybridization buffer. Then, the slice was washed in graded SSC buffer (2 ×, 1 ×, and 0.1 ×) for 30 min at 55°C and blocked with Blocking Reagent (Roche Diagnostics, Germany). DIG was detected with an alkaline phosphatase-conjugated anti-DIG antibody (Roche Diagnostics, Germany; diluted 1:1,000), and chromogenic development was conducted with NBT/BCIP Stock Solution (Roche Diagnostics, Germany).

### Static Incubation of Stomach and Intestinal Fragments and Brain Cells

The fresh stomachs and intestines were washed with phosphate buffer saline (PBS), and the tissues were cut into fragments (<1 mm^3^). All pieces were then evenly distributed in a 24-well culture plate with 1 ml of M199 medium (SparkJade, China) per well (containing double antibodies) and incubated at 27°C. After preincubation for 3 h, different concentrations of the conserved NPFF peptide (10^−6^ and 10^−7^ M) were added to the corresponding test well, and equal amounts of M199 medium were used as controls. Three replicates of each treatment were placed in a 27°C incubator for 1, 3, and 6 h. Experimental tissues were collected and stored at−80°C for the measurement of *gastrin* (*gas*) (MK825881), *ghrelin* (*ghrl*) (MH046053.1), *motilin* (*mtl*) (MH046054), and *cholecystokinin* (*cck*) (MK825882) expression after incubation.

Spotted sea bass brain cell culture was prepared according to the method described by Wong MK (49) and Yan-Horn Lee (50). The intact brains were quickly removed and cleaned in 10 mL of PBS. The isolated brain tissues were then transferred into 5 mL of fresh trypsin (SparkJade, China) and chopped into small pieces with a pair of scissors. After removing the trypsin by centrifugation, the cells were further pipetted up and down until dissociated. The mixture of dispersed brain cells and tissues was filtered, and the new M199 medium was added to the brain cells and mixed thoroughly. A 1 mL cell suspension was applied to the 24-well tissue culture plate and cultured for 3 days at 27°C. The static incubation and cell collection were performed as described above. After incubation, real-time PCR was performed for the detection of *orexin* (*orx*) (MK825880), *neuropeptide Y* (*npy*) (KJ850326.1), *leptin* (*lep*) (MK825878), *somatostatin* (*ss*) (MK825879), and *cck*. In contrast to mammals, *leptin* was found in the brains of several teleost, including green sunfish (51), grouper (52), goldfish (53), and medaka (54). Before evaluating the effect of the conserved NPFF peptide on the genes mentioned above, we measured their expression in the cultured cells (data not shown).

### Quantitative Real-Time PCR (qRT-PCR)

The levels of all tested genes were measured using qRT-PCR assays. The StepOne Plus Real-Time PCR system (Applied Biosystems, USA) was used to conduct qRT-PCR, and the 2^−ΔΔCT^ method was used to analyse the expression levels of genes. Every primer used in qRT-PCR is listed in Table 1, and the optimal cDNA concentration was determined by comparing the Ct values of the standard curves. qRT-PCR was performed using TB Green™ II Premix Ex Taq™ GC (Perfect Real Time) (Takara, Japan) with a reaction mixture containing 10 μL of TB Green™ Premix (2 **×**), 2 μL of template, 6.8 μL of sterilized distilled water, 0.4 μL of ROX and 0.4 μL of each forward and reverse primer. The template was amplified at 95°C for 30 s, followed by 40 cycles of 95°C for 5 s, 55°C for 30 s and extension at 72°C for 30 s. The *18S* rRNA gene was used as the internal reference for qRT-PCR normalization.

### Statistical Analysis

All data are shown as the mean ± SEM. Statistical analysis was carried out using SPSS software version 20.0. One-way ANOVA followed by Duncan's multiple range test and Fisher's least significance difference (LSD) test was used to identify significant differences. Any difference with a *P* < 0.05 was deemed significant.

## Results

### Gene Cloning and Sequence Analysis of Spotted Sea Bass *npff* and *npffr*s

**Figure 1:**
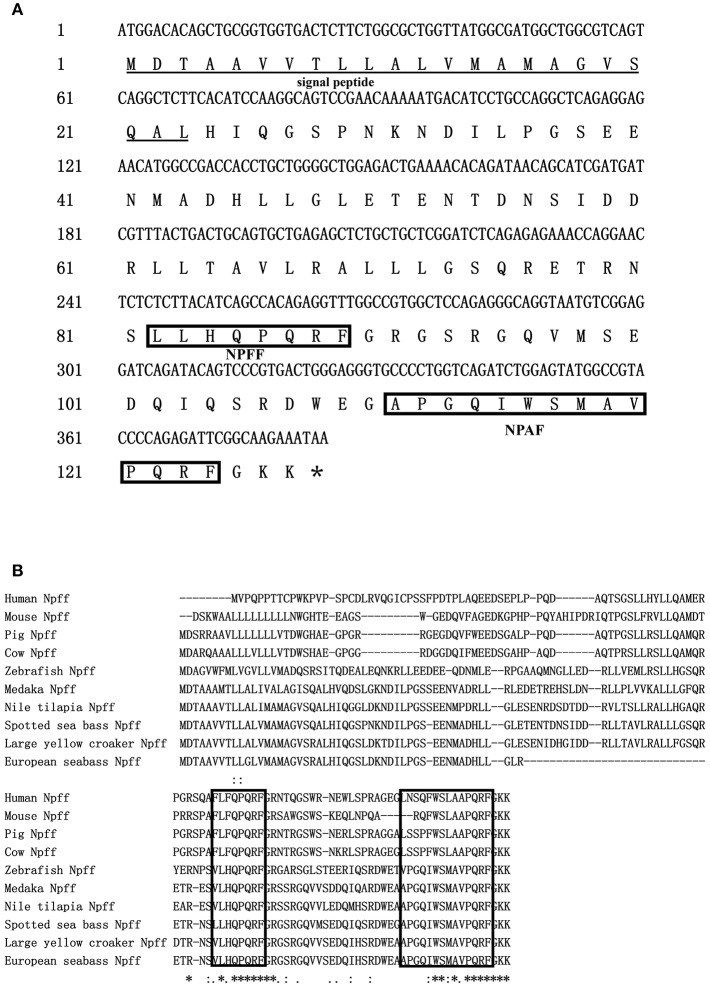
Sequence analysis of *npff* in spotted sea bass. **(A)** Nucleotide and deduced amino acid sequences of *npff* in spotted sea bass. The asterisks represent the stop codons. Single underlines represent signal peptides. The predicted conserved peptides of NPFF and NPAF of spotted sea bass are boxed. **(B)** Amino acid sequence alignment of spotted sea bass Npff with other vertebrates.

*npff* and *npffr*s were cloned via RT-PCR using spotted sea bass brain RNA as the template. The spotted sea bass *npff* cDNA sequence has an ORF of 384 bp and encodes a protein of 127 amino acids (Figure 1A). The NPFF precursor has a predicted signal peptide of 23 amino acids and contains two mature RF-amide peptides (NPFF and NPAF). Based on the predicted cleavage and amidation site, two putative mature peptides are theoretically NPAF: DWEGAPGQIWSMAVPQRFa and NPFF: NSLLHQPQRFa in the spotted sea bass. However, further purification and identification are still needed to confirm the exact mature peptides sequences. The spotted sea bass NPFF precursor has a low homology to those of humans and rats but a higher homology to those of other teleosts (Figure 1B) compared with the amino acid sequences of other species. However, two mature peptides are highly conserved compared with other species (Figure 1B). Phylogenetic analyses revealed that the spotted sea bass NPFF precursor remains largely conserved in other vertebrates and grouped closely with those of other teleosts (Figure 4A). The amphioxus PQRFa precursor was used as an out group.

**Figure 2:**
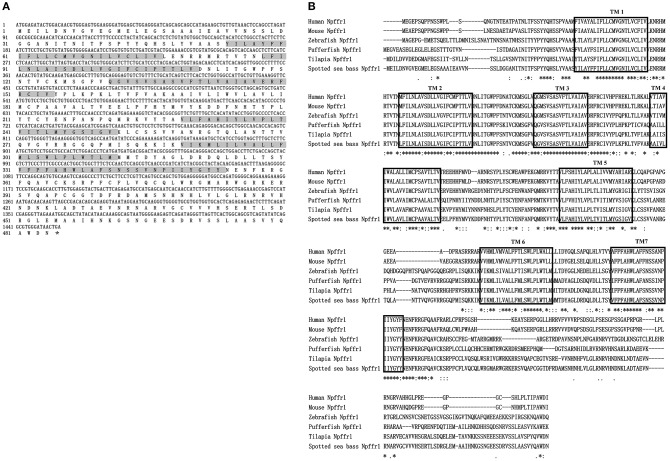
Sequence analysis of spotted sea bass *npffr1*. **(A)** Nucleotide and deduced amino acid sequences of *npffr1* in spotted sea bass. The stop codon is marked by an asterisk; shading represents transmembrane domains. **(B)** Comparison of the spotted sea bass Npffr1 sequences with those of other species. The transmembrane domains are boxed.

**Figure 3:**
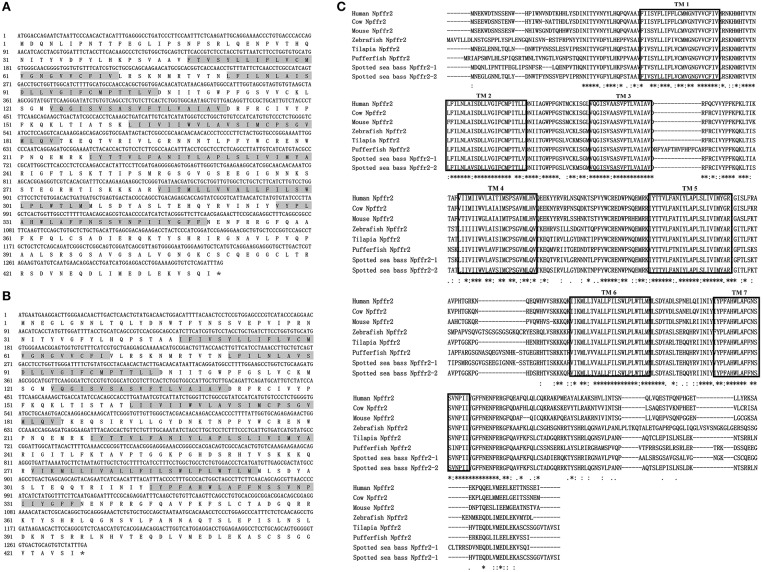
Sequence analysis of spotted sea bass *npffr2-1* and *npffr2-2*. **(A,B)** Nucleotide and deduced amino acid sequences of *npffr2-1* and *npffr2-2* in spotted sea bass. The stop codon is marked by an asterisk; the transmembrane domains are represented by shading. **(C)** Comparison of the spotted sea bass Npffr2-1 and Npffr2-2 sequences with those of different vertebrates. The conserved transmembrane domains of all species are boxed.

**Figure 4:**
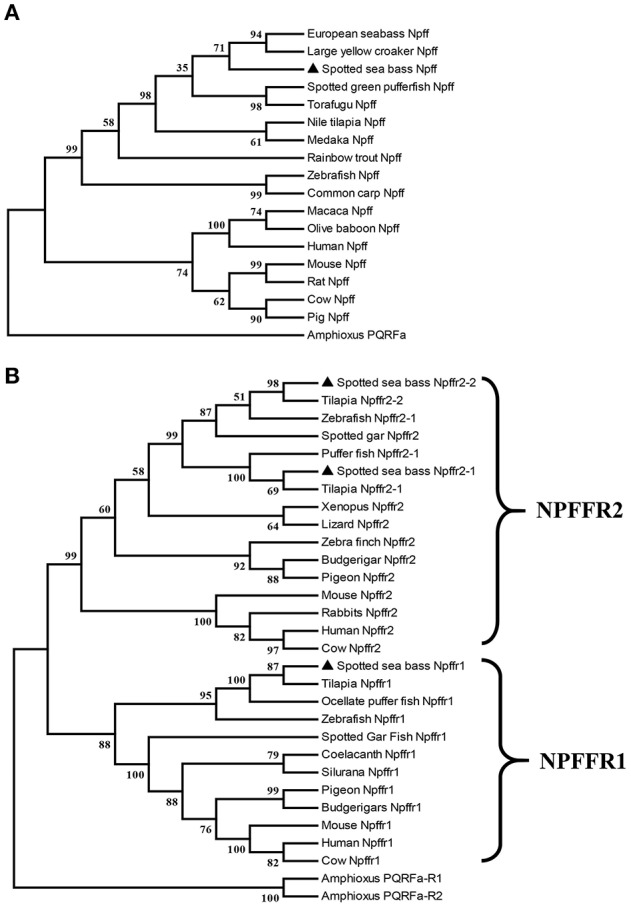
Phylogenetic analysis of the **(A)** Npff and **(B)** Npffrs sequences of spotted sea bass. The phylogenetic tree was constructed by MEGA 6 software using the neighbor-joining (NJ) method with 1000 bootstrap replicates. The number shown at each branch indicates the bootstrap value (%). Npff and its receptors are marked using (▴). The accession numbers of all NPFF amino acid sequences used for phylogenetic analysis are Human (AAI04235.1), Mouse (AAD39829.1), Rat (NP_072108.1), Pig (XP_003126243.1), Cow (AAD39827.1), Chicken (XP_015155812.1), Zebrafish (BAF34891.1), Medaka (XP_004068716.1), Nile tilapia (XP_003451891.1), Large yellow croaker (XP_010754783.1), European sea bass (CBN80856.1), Rainbow trout (XP_021426188.1), Common carp (XP_018967873.1), Spotted green pufferfish (BAF34885.1), Torafugu (NP_001092116.1), Macaca (XP_005571091.1), Olive baboon (XP_021778155.1) and Amphioxus (AB863739); those of the NPFFR1 amino acid sequences are Human (NP_071429.1), Mouse (NP_001170982.1), Cow (XP_027386149.1), Zebrafish (NP_001082858.1), Tilapia (XP_019220720.1), Ocellate puffer fish (NP_001092117.1), Spotted gar (XP_015201730.1), Coelacanth (XP_005991458.1), Silurana (XP_004915843.1), Pigeon (XP_021139217.1), Budgerigars (KQK76167.1); and those of the NPFFR2 amino acid sequences were Human (NP_444264.1), Mouse (NP_573455.2), Rabbit (XP_017202989.1), Cow (XP_005208183.1), Zebrafish (XP_021332202.1), Tilapia (XP_003449675.1 and XP_005451602.1), Spotted gar (XP_015194438.1), Pufferfish (NP_001092119.1), Xenopus (XP_002940397.1), Lizard (XP_008110384.1), Zebra finch (XP_002187367.2), Budgerigar (XP_005150597.1), Pigeon (XP_005504341.1), Amphioxus PQRFa-R1 (AB863740) and Amphioxus PQRFa-R2 (AB863741).

The ORFs of *npffr1* (Figure 2A), *npffr2-1* (Figure 3A), and *npffr2-2* (Figure 3B) encode 484, 440 and 426 amino acids, respectively. Three NPFF receptors comprise all 7 transmembrane structures according to analysis using TMHMM Server 2.0 (http://www.cbs.dtu.dk/services/TMHMM/). The predicted transmembrane results were shown in Figure S1. The spotted sea bass Npffr2-1 and Npffr2-2 amino acid sequences are highly similar (73%) to each other, and Npffr1 is highly similar to Npffr2-1 (59%) and Npffr2-2 (58%). The transmembrane domains of spotted sea bass Npffrs (Figures 2B, 3C) are highly conserved in comparison to those of other mammals and teleosts. The amino acid sequences of Npffrs were highly homologous (55–97%) to those of other species, especially the Nile tilapia and spotted garfish. Phylogenetic analysis showed that Npffrs are clustered into two separate clades, the NPFFR1 and NPFFR2 amino acid clades. Three NPFF receptors are most closely related to those other teleosts, especially Nile tilapia, with high bootstrap values (Figure 4B). Amphioxus PQRFa-R1 and R2 were used as an out group.

### Functional Characterization of the Spotted Sea Bass NPFF/Npffrs System in Cultured Eukaryotic Cells

**Figure 5:**
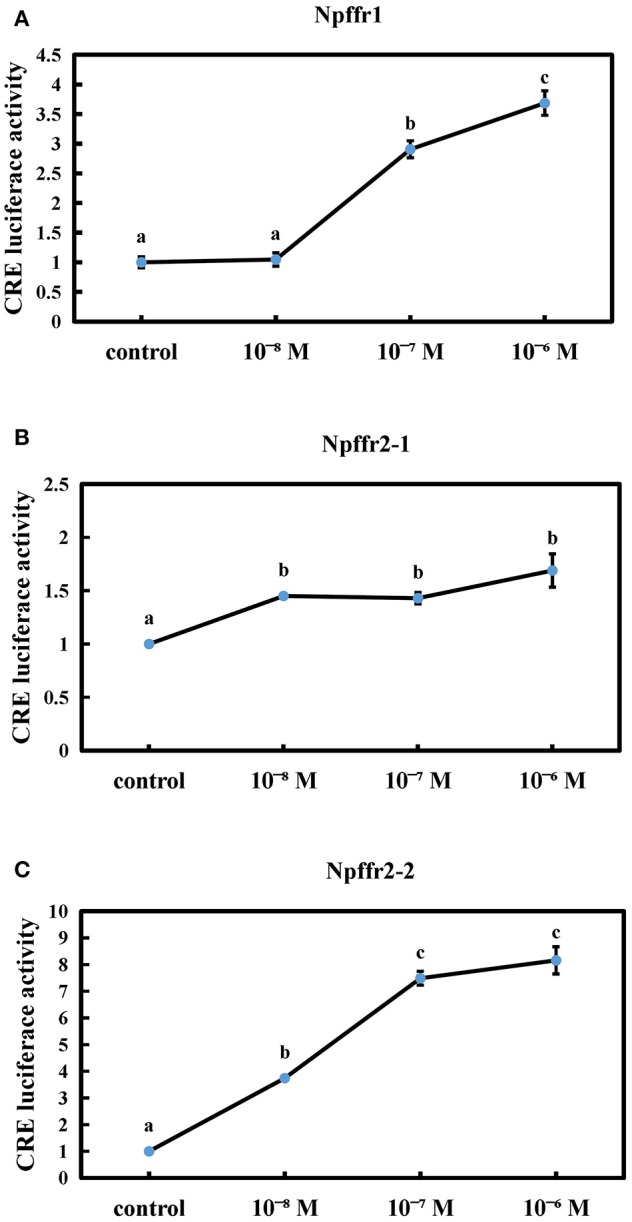
Functional interaction between the conserved NPFF peptide and Npffrs. Induction of CRE-driven luciferase activities by NPFF in 293T cells transfected with Npffr1 **(A)**, Npffr2-1 **(B)**, and Npffr2-2 **(C)**. The results are presented as the mean value ± S.E.M. from three independent experiments, each conducted in triplicate, and are expressed as the ratio of the increase in luciferase activity relative to that of the control.

We next tested whether the spotted sea bass conserved NPFF peptide can functionally interact with receptors using CRE reporter assays. The conserved NPFF peptide significantly increased the luciferase activity in cells transfected with spotted sea bass Npffr1 (Figure 5A), Npffr2-1 (Figure 5B), and Npffr2-2 (Figure 5C) in the CRE promoter assay. In the Npffr1 transfection experiments, CRE promoter activity was increased 3-fold in the 10^−6^ M NPFF group. Moreover, both the 10^−7^ and 10^−6^ M conserved NPFF peptides increased the luciferase activity of CRE to levels seven and eight times higher than that of the control group in the Npffr2-2 transfection experiments, respectively.

### Distribution of *npff* and *npffr*s mRNA in Different Spotted Sea Bass Tissues

**Figure 6:**
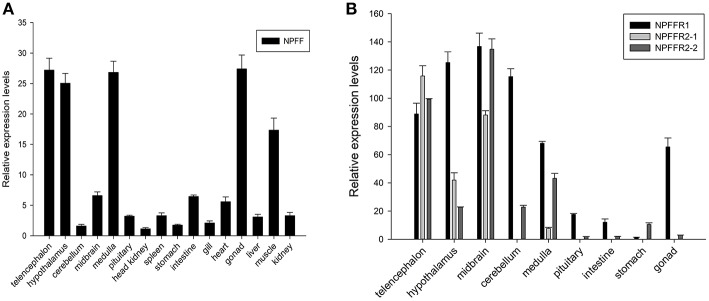
Relative expression levels of the **(A)** *npff*, **(B)** *npffr1, npffr2-1* and *npffr2-2* genes in different tissues of spotted sea bass. In this study, the brain regions were divided into the telencephalon, hypothalamus, cerebellum, midbrain, medulla, and pituitary. The expression levels of the four genes were quantified by qRT-PCR and normalized by *18S* rRNA. All data are presented as the mean ± S.E.M. (*n* = 3).

Spotted sea bass *npff* and *npffr*s relative mRNA levels were detected in various tissues via quantitative real-time PCR (qRT-PCR). As shown in Figure 6A, *npff* mRNA was widely expressed in the CNS and peripheral nervous system (PNS), and the highest expression occurred in the telencephalon, hypothalamus, medulla, gonad, and muscle. The *npff* mRNA expression in the intestines was slightly higher than that in other peripheral tissues. The *npffr*s mRNA expression was mainly distributed within the central nervous tissues compared to that in peripheral nervous tissues (Figure 6B). The expression of *npffr2-1* was considerably high in the telencephalon, midbrain, hypothalamus and medulla, notably in the telencephalon and midbrain, but not in other tissues. The highest expression levels of *npffr1, npffr2-1*, and *npffr2-2* were detected in the midbrain, telencephalon and midbrain, respectively. In the stomach and intestine, the expression levels of *npffr1* and *npffr2-2* mRNA were relatively low, but their actual expression levels were not low according to the observed Ct value and agarose gel electrophoresis results (data not shown).

### *npff* mRNA Cellular Localization in the Spotted Sea Bass Brain

**Figure 7:**
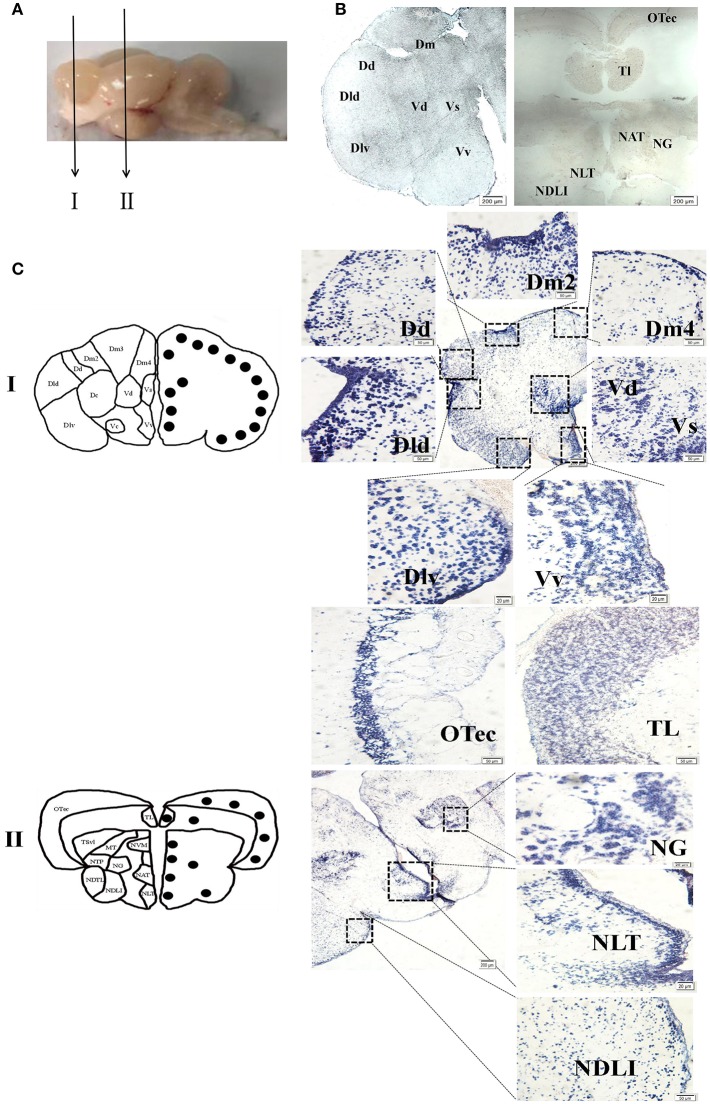
Localization of *npff* mRNA-expressing cells in the spotted sea bass brain. **(A)** Schematic illustrations indicating the positions of the coronal drawings and brain slides selected for **(B)** and **(C)**. **(B)** Sense probe results of *in situ* hybridization within the telencephalon, hypothalamus and midbrain regions of spotted sea bass. Scale bars = 200 μm. **(C)** Antisense riboprobes results at the same positions as those shown in **(B)**. The brain region nomenclature is marked in the micrograph on the left side, and the black dots represent the position of *npff*. On the right side, the distribution of *npff* mRNA-expressing cells in different regions of the brain is represented by blue dots. Scale bars = 50 μm.

To evaluate the function of spotted sea bass NPFF, based on the relative *npff* mRNA expression, the cellular localization of *npff* mRNA was further detected in the spotted sea bass brain (telencephalon, midbrain, hypothalamus) via *in situ* hybridization (ISH). Abbreviations for the telencephalon, hypothalamus, and midbrain region denominations are listed in Table 2. The schematic illustration in Figure 7A was to show the positions of the slides where *npff* mRNA was detected within the brain. As shown in Figure 7B, no signals were found in the telencephalon, midbrain or hypothalamus using a sense probe. The results of ISH were generally consistent with those of the quantitative analysis. In brief, *npff* mRNA was widely distributed in the telencephalon, midbrain and hypothalamus of adult spotted sea bass. The hybridization signals observed in the telencephalon were higher than those in other sections. Cells expressed *npff* at the highest levels in the ventralis telencephali pars supracommissuralis and pars ventralis (Vs and Vv) (Figure 7C). The cells of the dorsalis telencephali lateralis ventralis (Dlv), pars lateralis dorsal (Dld), pars dorsalis (Dd), pars medialis (Dm), and pars dorsalis (Vd) exhibited high *npff* mRNA expression (Figure 7C).

**Table 2:** Abbreviations for the telencephalon, hypothalamus, and midbrain region denominations in spotted sea bass.

**Abbreviation**	**Full name**
Dc	Area dorsalis telencephali pars centralis
Dd	Area dorsalis telencephali pars dorsalis
Dld	Dorso-lateral zone of dorsal telencephalon
Dlv	Latero-lateral zone of dorsal telencephalon
Dm	Area dorsalis telencephali pars medialis
NAPV	Nucleus anterioris periuentricularis
NAT	Nucleus anterior tuberis
NDLI	Nucleus diffusus lobi inferioris
NG	Nucleus glomerulosus
NLT	Nucleus lateral tuberis
Npo	Nucleus preopticus
NPPV	Nucleus ventral periventricular pretectal
NVM	Nucleus ventromedialis thalami
OTec	Optic tectum
Tl	Longitudinal tori
Vd	Area ventralis telencephali pars dorsalis
Vs	Area ventralis telencephali pars supracommissuralis
Vv	Area ventralis telencephali pars ventralis

In Figure 7C, a noticeable number of *npff* mRNA-expressing cells were detected in the optic tectum (OTec), nucleus anterior tuberis (NAT) and lateral tuberis (NLT), followed by the nucleus diffusus lobi inferioris (NDLI) and the nucleus ventromedialis thalami (NVM) in the midbrain. *npff* mRNA-containing cells were concentrated in the OTec, NAT and NLT but scattered in the NDLI, which was related to the structures of the midbrain and hypothalamus containing high-density cells.

### NPFF *in vitro* Action on Genes Associated With Feeding in Spotted Sea Bass

#### *In vitro* Functional Analysis of the Conserved NPFF Peptide on the Expression of orx, npy, lep, ss and cck in Spotted Sea Bass Brain Cells

To further evaluate the effect of conserved NPFF peptide on spotted sea bass feeding regulation, static incubation of brain cells, intestine and stomach was performed.

**Figure 8:**
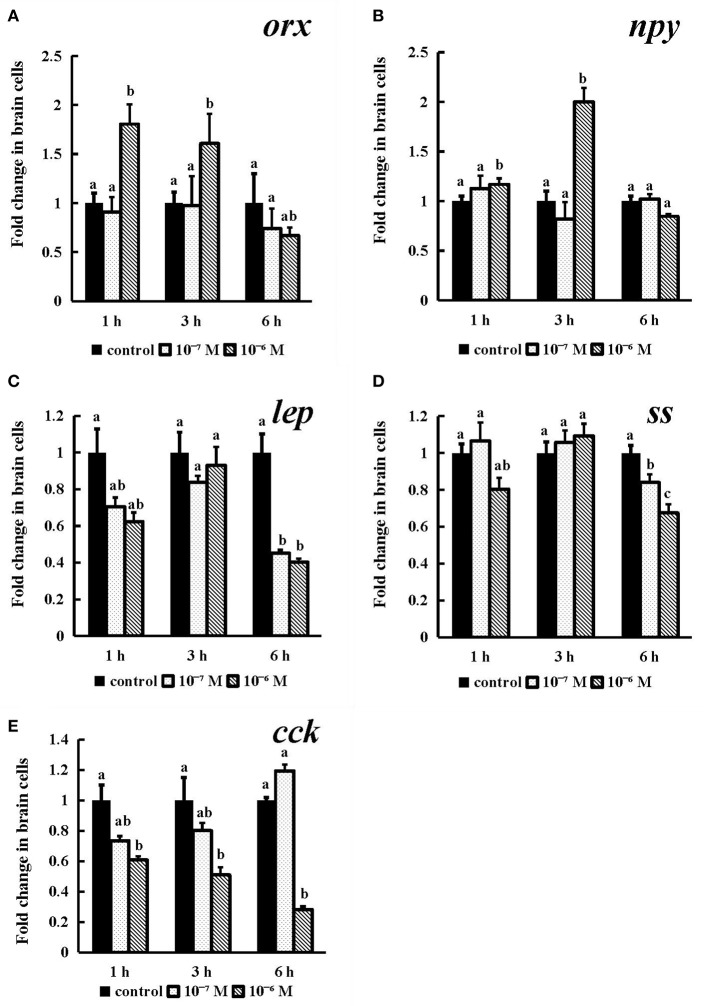
Effect of conserved NPFF peptide incubation on spotted sea bass *orx* **(A)**, *npy* **(B)**, *lep* **(C)**, *ss* **(D)**, and *cck* **(E)** mRNA expression in brain cells. Fold changes in the mRNA expression levels were studied in batch incubations in which three groups of brain cells were incubated for 1, 3, and 6 h in different concentrations (10^−6^ and 10^−7^ M) of NPFF relative to the mRNA expression in the normal incubation group without NPFF peptide treatment. The results are presented as the mean ± SEM (*n* = 3). Significant differences (*P* < 0.05) are noted by different letters in each concentration compared to the control. qRT-PCR was used to quantify the mRNA expression levels and normalized against *18S* rRNA.

As shown in Figure 8, the expression of *orx* was obviously increased after incubation for 1 h and 3 h (*P* < 0.05), and both exhibited the highest levels in the 10^−6^ M group (Figure 8A). 10^−6^ M conserved NPFF peptide could significantly heighten *npy* mRNA expression (P < 0.05) at 3 h post incubation (Figure 8B) and lower the levels of *lep* and *ss* mRNA (P < 0.05) in both tested doses at 6 h (Figures 8C, D), respectively. The expression of *cck* mRNA was sinificantly decreased (*P* < 0.01) in the 10^−6^ M group at all tested times (Figure 8E). These results indicated that all tested genes were sensitive to the treatment of the conserved NPFF peptide.

#### *In vitro* Effects of the Spotted Sea Bass Conserved NPFF Peptide on gas, ghrl, mtl, and cck mRNA Expression in the Intestine

**Figure 9:**
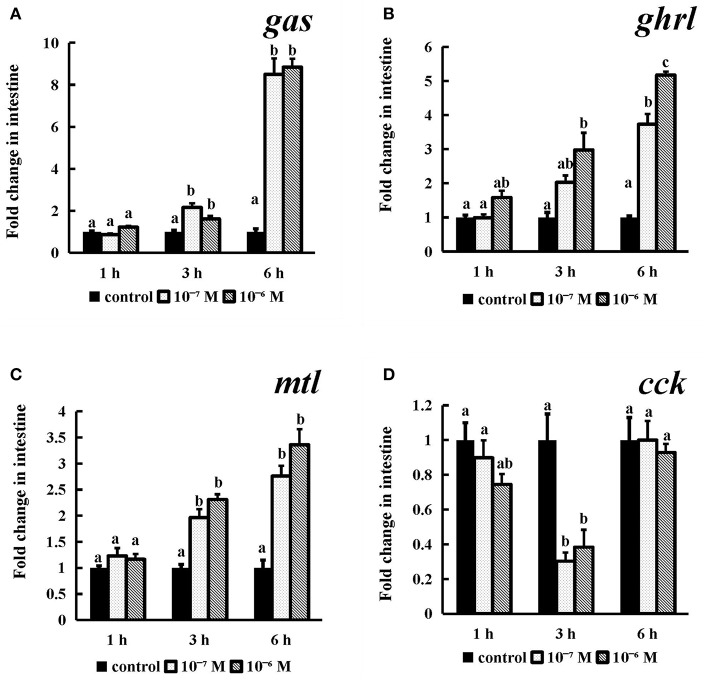
Effect of conserved NPFF peptide incubation on spotted sea bass *gas* **(A)**, *ghrl* **(B)**, *mtl* **(C)**, and *cck* **(D)** mRNA expression in the intestine. The results are presented as the mean ± SEM (n = 3) and normalized by *18S* rRNA. Significant differences (*P* < 0.05) are noted by different letters over each concentration compared to the control.

The *gas, ghrl, and mtl* mRNA expression (Figures 9A–C) levels were obviously dose- or time-dependently increased after conserved NPFF peptide incubation. Briefly, as time progressed, two tested doses conserved NPFF peptides significantly increased the *gas, ghrl* and *mtl* levels (*P* < 0.01). but had no significant variation at 1 h. In contrast, significant decreases in *cck* (*P* < 0.01) mRNA levels were observed at 3 h, and there were no significant differences between the two doses (Figure 9D).

#### *In vitro* Effects of the Spotted Sea Bass Conserved NPFF Peptide on gas, ghrl, mtl, and cck mRNA Expression in the Stomach

**Figure 10:**
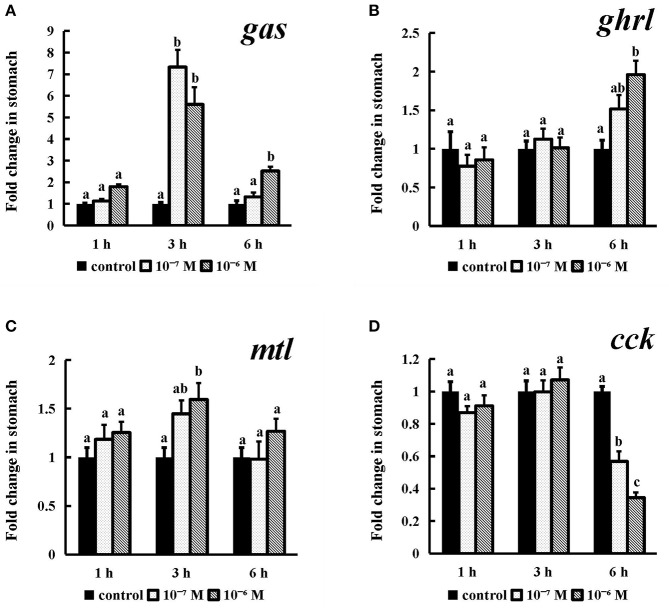
Effect of conserved NPFF peptide incubation on spotted sea bass *gas* **(A)**, *ghrl* **(B)**, *mtl* **(C)**, and *cck* **(D)** mRNA expression in the stomach. The results are presented as the mean ± SEM (*n* = 3). Significant differences (*P* < 0.05) are noted by different letters in each concentration compared to the control.

Incubation of conserved NPFF peptide significantly enhanced the expression levels of *gas* and *mtl* (Figures 10A,C) (*P* < 0.05) in stomach fragments at 3 h, but this effect disappeared thereafter. The *ghrl* mRNA expression levels (*P* < 0.05) were significantly increased at the longest tested incubation time (Figure 10B) at both of the tested concentrations relative to those in their control groups. Similarly, *cck* was also obviously expressed at a lower level than that in the control group after 6 h of incubation with 10^−7^ M and 10^−6^ M conserved NPFF peptide (*P* < 0.05) (Figure 10D). There were no significant differences in the rest of the experiments.

## Discussion

Increasing evidence has shown that the NPFF peptide can inhibit the appetites of rodents and agnathans by acting on the hypothalamic nucleus (34, 35, 55, 56). Nevertheless, studies on NPFF have merely involved molecular cloning and tissue expression in a few basal vertebrates (26, 28). The main purpose of this study was to initially to determine the role of the NPFF peptide in spotted sea bass feeding regulation.

The *npff* gene and three *npffr*s genes from spotted sea bass were cloned and characterized. The full-length sequence of *npff* is 384 bp, encoding a predicted protein of 127 amino acids with only one putative mature NPFF peptide. The similarity of the NPFF precursor amino acid sequence between spotted sea bass and humans was only 45%, but higher homologies (73–89%) were observed in comparison to several other bony fish, such as European seabass, medaka, Nile tilapia, and large yellow croaker. This finding is consistent with the results of the phylogenetic analysis. However, the putative mature peptide sequences were highly conserved in vertebrates, implying a conserved function during evolution. On the other hand, the obtained NPFF receptor sequences were all GPCRs sharing conserved seven transmembrane domains. Previous studies have identified and characterized the two *NPFFR*s in humans and rats (13); however, one *npffr1* gene and two *npffr2* genes were found in the spotted sea bass, which may be the result of a teleost-specific genome duplication event (57–59). Phylogenetic analysis showed that Npffrs are clustered into two separate clades (NPFFR1 and NPFFR2). Based on the above evidence, the four sequences we obtained were the spotted sea bass *npff* and *npffr*s.

Since the C-terminal plays an important role for the binding and activation of their receptors (60), conserved peptide of NPFF were synthesized. To test the binding activity of the conserved NPFF peptide to the three receptors, a cell line luciferase assay was performed, revealing that the synthetic spotted sea bass conserved NPFF peptide was capable of activating Npffrs to trigger downstream postreceptor events. It is worth noting that the binding ability of NPFF to Npffr2-2 was twice that of Npffr1. Previous reports have clarified that NPFFR1 (GPR147) and NPFFR2 (GPR74) can be both bind by the GnIH and NPFF in human, although NPFF preferentially activates NPFFR2 (61), which is also consistent with our results. Thus, spotted sea bass NPFF can exert physiological functions via Npffrs in different tissues.

Tissue distribution analysis showed that spotted sea bass *npff* mRNA was widely expressed in all tested tissues, with the highest expression observed in the telencephalon, hypothalamus, and medulla in the CNS and in gonad and muscle in the peripheral tissues. However, in rats, the highest level of *npff* mRNA was found in the spinal cord, pituitary and hypothalamus (62, 63), while the highest level in humans was observed in the medulla and spinal cord (64). Different expression patterns indicate variation in brain function among species. The highest levels of *npff* receptor mRNA in spotted sea bass were mainly in the CNS, including the telencephalon, hypothalamus, cerebellum, midbrain and medulla. These results were similar to the observations in mammals (13). In addition, in the present study, the expression level of *npffr1* was high in the intestine, and that of *npffr2-2* was high in the stomach. Taking the binding assay results together, both GnIH and NPFF were able to act on the food intake via NPFFRs in different organ with preference variations.

The cellular localization of *NPFF* in humans and rats was detected in many regions of the CNS. For example, human *NPFF* mRNA was localized in the brain and spinal cord (2). In rats (65), *NPFF* mRNA is highly expressed in the hypothalamic paraventricular nucleus (PVN), an autonomic nucleus critical for the secretion of neurohormones and the regulation of sympathetic outflow. Unlike in humans and rats, the localization of cells expressing *npff* mRNA have thus far been determined in only adult zebrafish and embryos (25). *zfPQRF*-expressing neurons were found in the olfactory bulb and nucleus olfactoretinalis in the telencephalon but were absent in the hypothalamus, brain stem and spinal cord. However, in our study, although the strongest signal was observed in limited areas of the telencephalon, such as the Vs and Vv, spotted sea bass *npff* mRNA was abundantly expressed in some areas of the hypothalamus, including the NDLI which is related to reproduction regulation (66), and the NAT, NLT, and NVM, which are related to feeding regulation (67–69). The differential localization of these genes between mammals and teleosts indicates differential gene functions. According to our results, both *npff* and its receptors were located in the hypothalamus, indicating a direct action of NPFF on hypothalamic neurons to regulate food intake (70).

Several studies have examined the biological functions of NPFF (PQRFa) in fish and basal vertebrates. In dwarf gourami (*Colisa lalia*), NPFF can inhibit the pacemaker activity of *TN-GnRH* neurons (27). Several studies have suggested that NPFF may play multiple roles in the reproductive cycle of grass puffers (32). There is also evidence that PQRFamide peptides may act as neuroregulators of at least the lamprey GnRH-II system in adult female lamprey (71). However, NPFF has not been reported to be involved in the regulation of fish feeding. For this purpose, primary brain cell culturing and static incubation in stomach and intestinal tissues were employed to assess the expression of feeding-related genes, including *orx, npy, lep, ss, gas, ghrl, mtl* and *cck*, after the conserved NPFF peptide stimulation. In rats, i.c.v of NPFF to the thalamic nucleus led to the apparent phenomenon of reduced food intake (35). NPFF exerted dual actions in the parabrachial nucleus to modulate food intake in rats (36). In addition, lacking of *NPFFR2* may lead to reduced adiposity and decreased food intake in both male and female mice but especially in males (37). The heritable variability of *NPFFR2* is closely associated with an enhanced obesity risk; therefore, NPFFR2 plays a key role in obesity predisposition (72). In contrast to rodents, human NPFF was found to inhibit catecholamine-induced lipolysis in human fat cells via NPFFR2 activation (37). The GnIH (RFRP-3), a paralog of NPFF, significantly stimulated food intake in chick (38, 39). While, it simultaneously decreased *Pomc* and increased *Npy* mRNA levels in female jerboas via NPFFR1 (GPR147) (40). When energy availability is limited, RFRP-3 can coordinate the process of feeding and sexual behavior with ovarian steroids in Syrian hamsters (*Mesocricetus auratus*) and other species (73). Accordingly, NPFFR1 and NPFFR2 were able to mediate the effect of both GnIH and NPFF in feeding regulation and energy metabolism. In the present study, the conserved NPFF peptide exerted an effect opposite of that observed in rats. The conserved NPFF peptide significantly increased the expression levels of *orx, npy, gas, ghrl*, and *mtl* mRNA in brain cells and gastrointestinal tissues, while *lep, ss*, and *cck* were reduced by peptide treatment at different concentrations and times. It was proven that *npy, orx* and *lep* are potent orexigenic and obese factors in fish (74, 75), while *cck* and *ss* produced primarily in the brain and gastrointestinal tract act as satiety signals to reduce food intake (76, 77), and *gas, ghrl* and *mtl* play important roles in promoting gastrointestinal motility and gastric acid secretion (78, 79). Accordingly, the spotted sea bass NPFF peptide may act in both central and peripheral tissues to increase food intake and play a negative role in lipid metabolism and obesity regulation by binding to Npffrs. Furthermore, these data indicate that the NPFF peptide may display marked differences amongst species in its ability to regulate feeding and lipolysis.

In summary, we cloned and identified the *npff* and *npffrs* cDNAs from spotted sea bass and, for the first time, functionally characterized the NPFF/Npffr system in a fish species in terms of sequence analysis, expression patterns, ligand-receptor interactions, *in situ* hybridization and *in vitro* physiological effects. Altogether, our data provide the first functional evidence that the NPFF peptide may play a stimulating role in feeding regulation in spotted sea bass. Our findings will lead to a better understanding of the NPFF/NPFFR system in the regulation of feeding processes in vertebrates.

## Data Availability

The raw data supporting the conclusions of this manuscript will be made available by the authors, without undue reservation, to any qualified researcher.

## Ethics Statement

All animal experiments were conducted in accordance with the guidelines and approval of the respective Animal Research and Ethics Committees of Ocean University of China.

## Author Contributions

HW, XQ, and YL designed the study. QL and ZZ performed in samples collection. QL and YZ performed the *in situ* hybridization experiment. QL performed the gene clone, static incubation, and qRT-PCR experiment. QL wrote the manuscript. XQ provided manuscript editing and feedback. All authors read and approved the final manuscript.

### Conflict of Interest Statement

The authors declare that the research was conducted in the absence of any commercial or financial relationships that could be construed as a potential conflict of interest.
