# Supplementary material for: Evidence for the Direct Effect of the NPFF Peptide on the Expression of Feeding-Related Factors in Spotted Sea Bass (Lateolabrax maculatus)
Source: Front Endocrinol (Lausanne). 2019 Aug 6;10:545. doi: 10.3389/fendo.2019.00545 (PMC6691130; doi:10.3389/fendo.2019.00545)
Supplement: Supplementary file 1 [file Image_1.pdf]

A

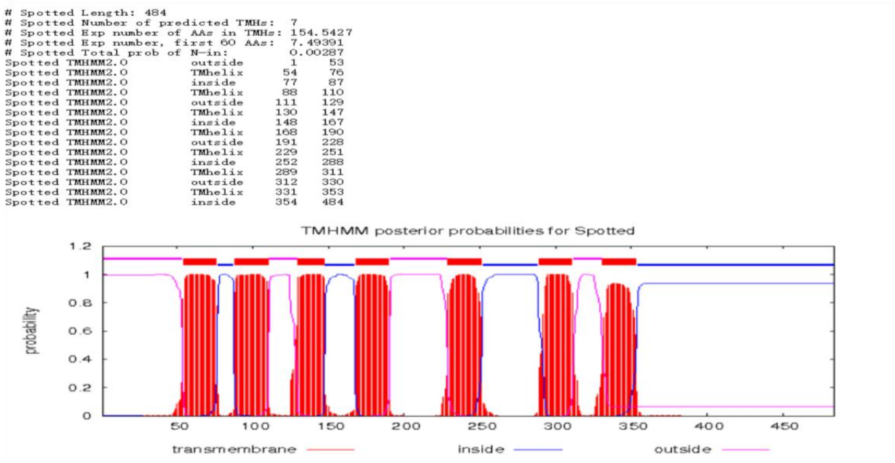

B

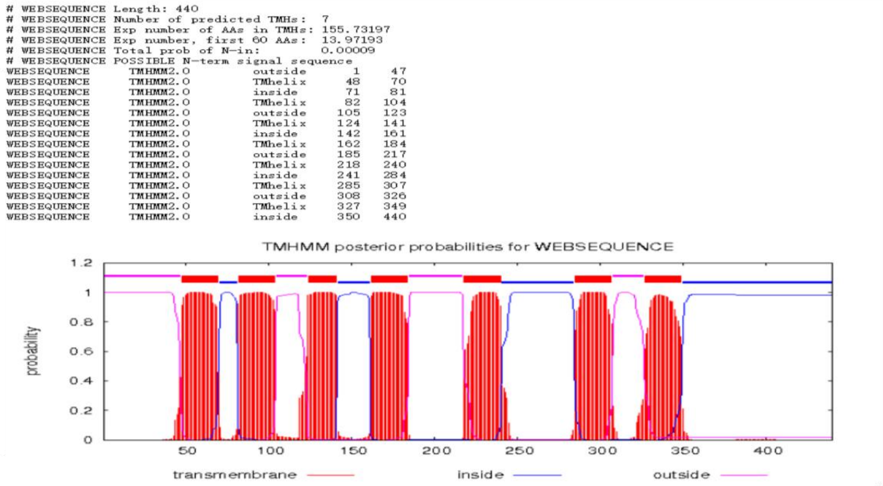

C

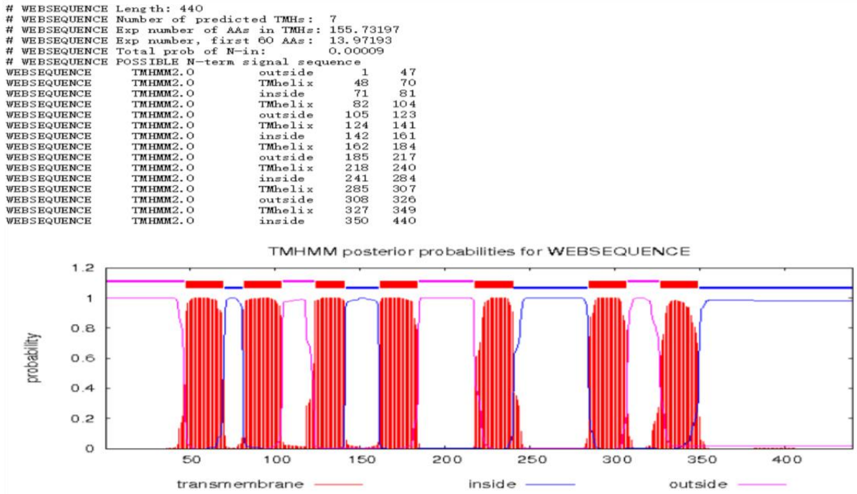

Fig.S1. Seven transmembrane region of NPFFR1, NPFFR2-1 and NPFFR2-2. (**A**) NPFFR1. Transmembrane region of the spotted sea bass amino acids sequence. (**B**) and (**C**) NPFFR2-1 and NPFFR2-2.
